# Mathematical Model of Innate and Adaptive Immunity of Sepsis: A Modeling and Simulation Study of Infectious Disease

**DOI:** 10.1155/2015/504259

**Published:** 2015-09-08

**Authors:** Zhenzhen Shi, Chih-Hang J. Wu, David Ben-Arieh, Steven Q. Simpson

**Affiliations:** ^1^Health Care Operations Resource Center, Department of Industrial and Manufacturing Systems Engineering, Kansas State University, 2037 Durland Hall, Manhattan, KS 66506, USA; ^2^Division of Pulmonary Diseases and Critical Care Medicine, University of Kansas, Mail Stop 3007, 3901 Rainbow Boulevard, Kansas City, KS 66160, USA

## Abstract

Sepsis is a systemic inflammatory response (SIR) to infection. In this work, a system dynamics mathematical model (SDMM) is examined to describe the basic components of SIR and sepsis progression. Both innate and adaptive immunities are included, and simulated results *in silico* have shown that adaptive immunity has significant impacts on the outcomes of sepsis progression. Further investigation has found that the intervention timing, intensity of anti-inflammatory cytokines, and initial pathogen load are highly predictive of outcomes of a sepsis episode. Sensitivity and stability analysis were carried out using bifurcation analysis to explore system stability with various initial and boundary conditions. The stability analysis suggested that the system could diverge at an unstable equilibrium after perturbations if *r*_*t*2max_ (maximum release rate of Tumor Necrosis Factor- (TNF-) *α* by neutrophil) falls below a certain level. This finding conforms to clinical findings and existing literature regarding the lack of efficacy of anti-TNF antibody therapy.

## 1. Introduction 

Sepsis, currently defined as a systemic inflammatory response (SIR) to an infectious agent or trauma, is increasingly considered an exaggerated, poorly regulated innate immune response to microbial products [1, 2]. Under health conditions, intruding pathogens are eliminated by immune cells in the immune system. If overwhelming immune response occurs, unbalanced responses between immune cells lead to unexpected harmful patient outcomes such as high fevers, flushed skin, and elevated heart rate, resulting in sepsis. Possible progression to severe sepsis is marked by generalized hypotension, tissue hypoxia, and* Organ Dysfunction* [1]. Severe sepsis can further develop into septic shock under long-lasting severe hypotension [3], ultimately leading to death.

Severe sepsis and septic shock during an infection are the primary causes of death in intensive care settings [4]. On average, sepsis causes 250,000 deaths per year in the United States [5]. Among patients in intensive care units (ICUs), sepsis is the second highest cause of mortality [6] and the 10th leading cause of death overall in the United States [7]. An average of 750,000 sepsis cases occur annually, and this number continues to increase each year [6]. Care of patients with sepsis can cost as much as $60,000 per patient, resulting in a significant healthcare burden of nearly $17 billion annually in the United States [8, 9]. Sepsis development in a hospitalized patient can lead to extended hospital stays and consequently increase financial burdens. Cross and Opal [10] pointed out the lack of rapid, reliable assays that could be used to identify the stage or severity of sepsis and to monitor the use of immunomodulatory therapy. However, no such assays are available because complexity of the inflammatory response and the unpredictable nature of septic shock in individual patients render the effect of targeting isolated components of inflammation with supportive therapy difficult to predict [10, 11].

Development of a nonbiased, predictive model and model-derived policies that prevent patients from experiencing severe consequences of sepsis (e.g.,* Organ Dysfunction*) is critical for improving ICU patient care. As studies of mechanisms leading to sepsis development significantly progress due to discoveries of new inflammatory proteins and increased knowledge of the interaction of host cells and pathogens, mathematical models have been developed as dynamic knowledge representation of complicated biological processes. Specifically, the models have been used to simulate dynamic patterns of selected essential indicators in disease progression by integrating cellular and molecular pathways in an immune system. These mathematical models offer potential for understanding complex dynamic systems and, therefore, are used by researchers from various fields to simulate immune response to specific disease [12–15]. Development of modeling techniques could allow novel strategies for disease treatment, oriented at compromising harmful effects of inflammatory responses, to be proposed or tested in model simulations.

In order to construct a mathematical model of sepsis, we searched literatures and found two representative system dynamics mathematical models (SDMMs) of Acute Inflammatory Response (AIR) in previous studies. In 2004, Kumar et al. [12] presented a simplified 3-equation SDMM to describe mathematical relationships between pathogen, early proinflammatory mediators, and late proinflammatory mediators in sepsis progression. In 2006, Reynolds et al. [13] proposed a mathematical model for AIR that included a time-dependent, anti-inflammatory response in order to provide insights into a variety of clinically relevant scenarios associated with inflammatory response to infection.

## 2. System Dynamics Mathematical Model Development

Existing mathematical models focused on inflammation in the literature proved that mathematical modeling is a valid approach for simulating disease progression [12–16]. However, the number of variables used, the limited control of system parameters, and the inclusion of many variables involved in real immune response were not modeled in detail. Therefore, oversimplification in AIR models [13, 15] limited AIR behaviors and biological relevance of simulated results. For example, simulated results from AIR models [13, 15] failed to capture a dampened oscillated infection in AIR progression. In addition, existing mathematical models are incomplete representations of sepsis because simulated AIR in both mathematical models [13, 15] is considered to be an initial stage of sepsis progression. Therefore, to improve on current models, we developed an 18-equation SDMM to incorporate the most influential variables for septic response development during innate immune response and adaptive immune response. We included equations to represent pathogen load, phagocyte (including neutrophil and monocyte) activation, early and late proinflammatory cytokine release, tissue damage, anti-inflammatory cytokine release, CD4+ T cell activation, CD8+ T cell activation, B cell activation, and antibody release. Indicator selection was based on knowledge of cellular and molecular pathways of sepsis from experts in the field and extensive literature review [4, 17–28]. We chose* Salmonella* as a “targeted” pathogen strain in our mathematical model and simulated immune responses to* Salmonella* in the liver of mice. Immune responses to* Salmonella* infections have been investigated extensively in [20, 29–33]; therefore, an abundance of data exists for accurate incorporation of relationships among variables to support our SDMM. We used a series of known and hypothesized kinetics of biological system components, including conventional logistics function, law of mass action, and Michaelis-Menten kinetics to build SDMM from subsystems and mimic interactions between indicators. We combined these formulated but generalized dynamic modeling techniques into a comprehensive SDMM framework to describe sepsis progression, by measuring the steady state of various components in inflammatory responses. In the following seven subsections, we present a detailed description of mathematical construction for each subsystem in a mouse hepatic inflammatory response during SDMM development.

### 2.1. Process Description

**Figure 1:**
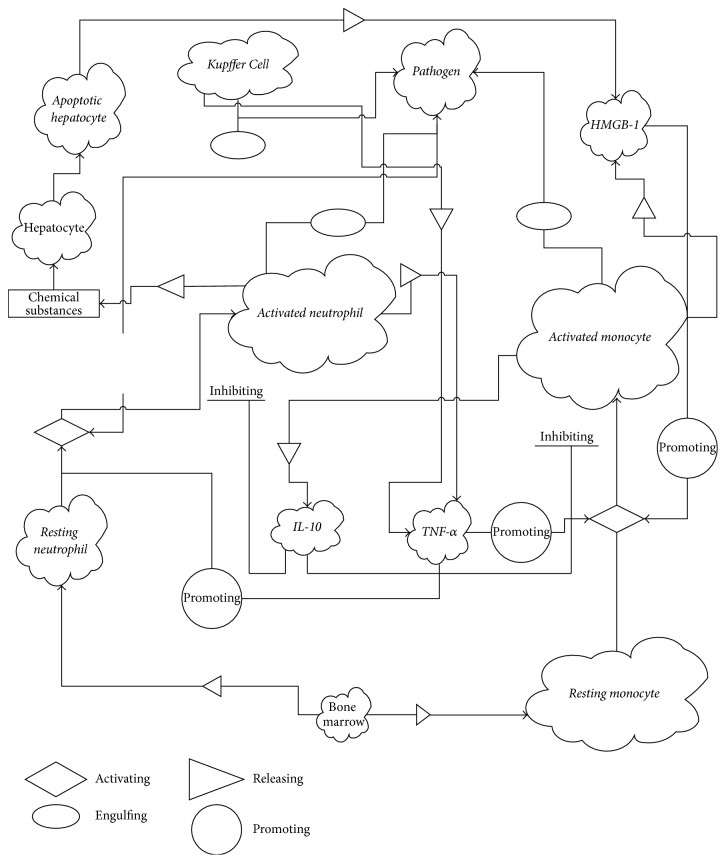
Types of indicators (cells and cytokines) and their interactions in AIR progression. Italic letters represent variables in our SDMM.

AIR typically occurs when immune cells, such as tissue macrophage, detect intruding pathogens or existing tissue damage and emit a signal to resting phagocytes, such as neutrophil and monocyte (two types of immune cells), in the blood vessels near the infected tissue. Resting phagocytes are activated and migrate towards the site of pathogens or damaged tissue that has recognizable proteins on surface similar to proteins of immune cells. Once activated phagocytes reach the infection site, they engulf and consume the pathogens. Meanwhile, these activated phagocyte cells release proinflammatory cytokines such as Tumor Necrosis Factor-*α* (TNF-*α*), Interleukin-1 (IL-1), Interleukin-6 (IL-6), Interleukin-8 (IL-8), and High-Mobility Group Protein B1 (HMGB-1) that activate and recruit additional resting phagocytes from circulation to the infection site. All activated phagocytes eliminate pathogens and secrete substances that accelerate the killing of healthy cells and induce inflammation in the initial process of inflammatory response. In the later stage of AIR progression, several types of anti-inflammatory mediators, such as Interleukin-10 (IL-10), are released by activated phagocytes (primarily monocyte-derived-macrophage). These anti-inflammatory cytokines inhibit the production of proinflammatory cytokines, consequently inhibiting further recruitment of resting phagocytes. We translated biological processes of AIR to a logical chart, as shown in Figure 1. An explicit description for each biological process is presented in the following six subsections.

### 2.2. Step 1: Kupffer Cell Local Response Model

Macrophages, one of the innate host's first lines of defense against bacterial pathogens, are antimicrobicidal cells that often determine outcomes of an infection [21]. Furthermore, hepatic macrophages (also known as Kupffer Cells or resident liver macrophages) constitute 80%–90% of tissue resident macrophages in the body and significantly influence propagation of liver inflammation [34, 35]. Kupffer Cells within the liver trap and eliminate a majority of bacteria that enter the blood stream [22]. During the initial stage of AIR, Kupffer Cells eliminate pathogens, specifically* Salmonella*, during local immune responses.

We developed a Kupffer Cell local response model, defined as interactions between the pathogen and Kupffer Cell [34], consisting of the following:

(1) dPdt=kpgP1−PP∞−rpmkPnPn+kc1nMkfP∗,

(2) dMkfdt=kmkMkf1−MkfK∞+kmkubMkb−PnPn+kc1nMkfP∗−umkMkf,

(3) dMkbdt=PnPn+kc1nMkfP∗−kmkubMkb.

In (1), *P* denotes pathogen load, *k*_pg_ represents a constant growth rate for pathogens, and *P*_*∞*_ represents maximum carrying capacity of the pathogen. Parameter *r*_*pmk*_ represents phagocytosis (killing) rate of Kupffer Cells when Kupffer Cells begin to kill pathogens. Although phagocytosis rate is dependent on time in a slow-S-shape curve [23], the phagocytosis rate does not change if the phagocytosis rate versus time is assumed to be linear. Therefore, we relaxed the condition that phagocytosis rate is constant in our model and assumed *r*_*pmk*_ was constant [23]. Equation (2) represents changes of Kupffer Cells over a unit of time, and *M*_*kf*_ denotes the amount of Kupffer Cells in the liver that is available for pathogen binding. Parameter term *k*_*mk*_ represents a constant proliferation (replenishment) rate for Kupffer Cell population, and *K*_*∞*_ represents maximum carrying capacity of Kupffer Cells in the liver of mice. Parameter term *k*_*mkub*_ represents the unbinding rate of binding Kupffer Cells and *u*_*mk*_ represents the killing rate of free Kupffer Cells induced by binding to intruding pathogens.

A standard logistic function was used to model pathogen population growth with limited maximum carrying capacity, identified as the first term (*k*_pg_ *P*(1 − *P*/*P*_*∞*_)) in (1) [36]. The second term of (1) models local Kupffer Cell responses or decrease in pathogen population phagocytized by initial tissue resident macrophages (Kupffer Cells). The process of phagocytosis includes two steps: pathogen-ligand binding to receptors of Kupffer Cells and phagocytosis by Kupffer Cells. We used a Hill-type function and receptor-ligand kinetics to model the two basic steps [21, 30, 34, 37–39]. First, we defined the rate of pathogen binding to Kupffer Cells as a Hill-type function ([*P* ^*n*^]/[*P* ^*n*^ + *k*_*c*1_^*n*^]) in which *n* represents a strong affinity of pathogen binding to Kupffer Cells and *k*_*c*1_ is Kupffer Cell concentration that phagocytoses half the pathogens. Second, we modeled pathogen to Kupffer Cell receptors using receptor-ligand kinetics (([*P* ^*n*^]/[*P* ^*n*^ + *k*_*c*1_^*n*^])*M*_*kf*_ *P* ^*∗*^), where *P* ^*∗*^ represents pathogen concentration. We determined pathogen concentration using the number of pathogens divided by maximum carrying capacity of the pathogen (10^8^ cells in the liver of mouse [32]). The final variable to determine pathogen decrease was the phagocytosis rate of pathogens by Kupffer Cells (represented by *r*_*pmk*_) times the portion of pathogens binding to Kupffer Cells (([*P* ^*n*^]/[*P* ^*n*^ + *k*_*c*1_^*n*^])*M*_*kf*_ *P* ^*∗*^).

We assumed that Kupffer Cells population growth followed a standard logistic growth pattern with a constant proliferation (replenishment) rate, denoted as *k*_*mk*_, and a maximum carrying limit,* K*_∞_, represented by the first term (*k*_*mk*_ *M*_*kf*_(1 − *M*_*kf*_/*K*_*∞*_)) in (2). Because pathogen binding did not preclude phagocytosis of additional pathogens after completion of phagocytosis, we used receptor-ligand kinetics to model the release of Kupffer Cells from the binding-complex, represented by the second term (*k*_*mkub*_ *M*_*kb*_) in (2); *k*_*mkub*_ represents the rate by which* Salmonella* are phagocytosed by free Kupffer Cells and made available for additional interactions with* Salmonella*. The decreasing number of free Kupffer Cells was due to free Kupffer Cells binding to pathogen, described by the third term (([*P* ^*n*^]/[*P* ^*n*^ + *k*_*c*1_^*n*^])*M*_*kf*_ *P* ^*∗*^), and the natural decay of free Kupffer Cells represented by the fourth term (*u*_*mk*_ *M*_*kf*_) in (2). Free Kupffer Cells become binding Kupffer Cells once they bind to pathogen, as described by the first term (([*P* ^*n*^]/[*P* ^*n*^ + *k*_*c*1_^*n*^])*M*_*kf*_ *P* ^*∗*^) in (3). The second term (*k*_*mkub*_ *M*_*kb*_) in (3) measures decreasing (releasing) portion of binding Kupffer Cells. The definition of parameters and corresponding experimental data for each system parameter in Kupffer Cell local response model are summarized in Table 1 (refer to Appendix).

### 2.3. Step 2: Neutrophil Immune Response Model

**Figure 2:**
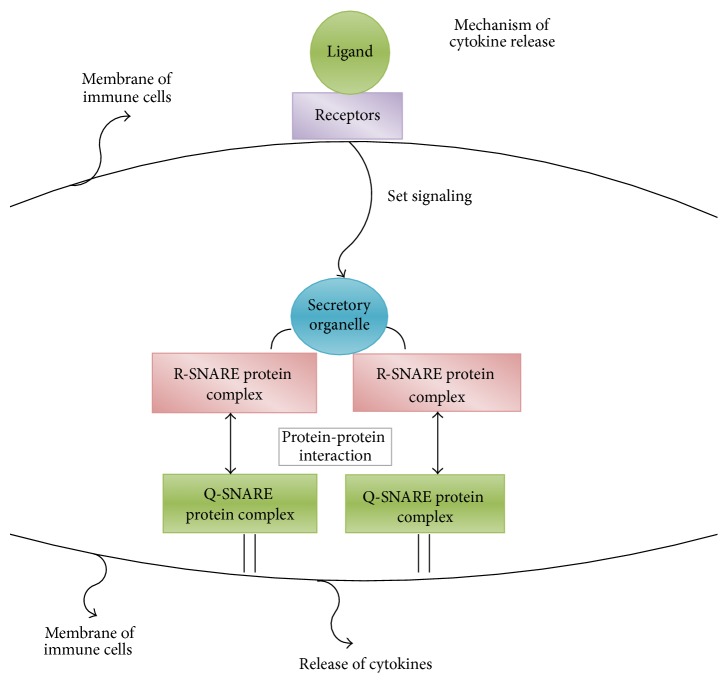
Mechanism of cytokine release.

Simulated results (data not shown) from our Kupffer Cell local response model indicated that Kupffer Cells may not sufficiently eliminate infection, especially when the local infection is overwhelming. Furthermore, pieces of evidence in biological studies have shown that recruitment of neutrophils (one type of immune cells) from circulation to the infection site significantly contributes to AIR progression because neutrophil is able to kill pathogens. Neutrophils accumulation is induced by a proinflammatory cytokine called “TNF-*α*” that is released by Kupffer Cells or activated neutrophils in the tissue. Release of cytokines follows trafficking machinery, and cytokines are released via protein-protein interactions initiated by ligand binding to receptors [61, 62]. The mechanism of cytokine release is depicted in Figure 2.

We modeled a protein-protein interaction as Michaelis-Menten kinetics [63] and derived our neutrophil immune response model as follows:

(4) dPdt=kpgP1−PP∞−rpmkPnPn+kc1nMkfP∗−rpnPnPn+kc2nNf+NbP∗,

(5) dMkfdt=kmkMkf1−MkfK∞+kmkubMkb−PnPn+kc1nMkfP∗−umkMkf,

(6) dMkbdt=PnPn+kc1nMkfP∗−kmkubMkb,

(7) dTdt=rt1max⁡Mkbmt1+MkbMkb+rt2max⁡Nbmt2+NbNb−utT,

(8) dNRdt=krdNR1−NRNS−r1NRT+P∗−μnrNR,

(9) dNfdt=r1NRT+P∗+knubNb−PnPn+kc2nNfP∗−μnNf,

(10) dNbdt=PnPn+kc2nNfP∗−knubNb,

(11) dr1dt=kr11+tanh⁡Nf∗−μr1r1.

Equation (4) was further derived from (1) in the Kupffer local immune response by incorporating phagocytic effects of neutrophils, represented by term *r*_*pn*_([*P* ^*n*^]/[*P* ^*n*^ + *k*_*c*2_^*n*^])(*N*_*f*_+*N*_*b*_)*P* ^*∗*^. Equations (5) and (6) are cited from (2) and (3).

Equation (7) represents changes of proinflammatory cytokines (denoted by* T*), such as TNF-*α*, released by binding tissue resident Kupffer Cells (*M*_*kb*_) and binding activated neutrophils (*N*_*b*_). Because TNF-*α* was released after pathogens bound to receptors of tissue resident Kupffer Cells or activated neutrophils, we modeled the process of TNF-*α* release as a combination of Michaelis-Menten kinetics and receptor-ligand kinetics [64]. In (7), the release of TNF-*α* from Kupffer Cells was initiated by receptor-ligand kinetics, followed by enzymatic kinetics (Michaelis-Menten), represented by the term (*r*_*t*1max⁡_ *M*_*kb*_/(*m*_*t*1_ + *M*_*kb*_)), where *r*_*t*1max⁡_ represents the maximum production rate of TNF-*α* by binding Kupffer Cells. The release of TNF-*α* is a combined effect of receptor-ligand kinetics and enzymatic kinetics; therefore, we incorporated both terms (*r*_*t*1max⁡_ *M*_*kb*_/(*m*_*t*1_ + *M*_*kb*_))*M*_*kb*_ in the model to represent combined effects of TNF-*α* releasing processes. Similarly, we used receptor-ligand kinetics and Michaelis-Menten kinetics to model the release of TNF-*α* from binding activated neutrophils in the second term of (7). The third term in (7), *u*_*t*_ *T*, measures degradation of TNF-*α*, with *u*_*t*_ representing the degradation rate of TNF-*α* per hour.

In (8), the first term *k*_*rd*_ *N*_*R*_(1 − *N*_*R*_/*N*_*S*_) is a standard logistic function to measure increase in the number of resting neutrophils per time unit (hour), represented by the influx of neutrophils into blood vessels per hour. The second term *r*_1_ *N*_*R*_(*T* + *P*)^*∗*^ indicates that the decrease in number of resting neutrophils per time unit is due to the neutrophils activation process promoted by pathogen and proinflammatory cytokine TNF-*α*, where *T* ^*∗*^ denotes concentration of TNF-*α* and *P* ^*∗*^ denotes concentration of pathogens [24, 65, 66]. The third term in (8), *μ*_*nr*_ *N*_*R*_, represents the natural decay of resting neutrophils; *u*_*nr*_ is defined as the apoptotic rate of resting neutrophils per time unit in hours. In (9), the first term exactly equals the second term in (8) because the increased population of activated neutrophils directly resulted from activation of the population of resting neutrophils. The second term of (9) used mass action kinetics (*k*_*nub*_ *N*_*b*_) to model the release of activated neutrophils from the binding-complex and make activated neutrophils available for additional interaction with pathogens, where *N*_*b*_ represents the binding-complex and *k*_*nub*_ represents the rate of activated neutrophils released from the binding-complex. Similar to the third term in (8), the third term of (9) models natural apoptosis of activated neutrophils. Equation (10) is similar to the derivation of (3) in the Kupffer local response model. We used a hyperbolic tangent function in (11) to represent a slow-saturation influx rate of neutrophils into hepatic parenchyma, thereby representing the rate of activated resting neutrophils. The definition and corresponding experimental data for newly added system parameters in the neutrophil immune response model are summarized in Table 2 (refer to Appendix).

### 2.4. Step 3: Damaged Tissue Model

Complexity in AIR progression is due to multiple effects induced by inflammatory cells. Recruitment of neutrophils helps clear local pathogen levels; however, those inflammatory cells are harmful because they release toxic molecules such as reactive oxygen species (ROS), which can damage host tissue [24, 66]. Recent experimental results have shown that neutrophils' *β*_2_ integrins adhere to ICAM-1 receptors of hepatocytes and accelerate the killing process of distressed hepatocytes [67].

We assumed the binding process of neutrophils to hepatocytes (healthy liver cells) also followed receptor-ligand kinetics; therefore, we derived the following damaged tissue model:

(12) $$\begin{array}{c} \frac{dD}{dt}=r_{hn}\frac{\left[D^{n}\right]}{\left[D^{n}+k_{c3}^{n}\right]}N_{f}D^{*}\left(\;1-\frac{D}{A_{\infty }}\;\right)-r_{ah}D. \end{array}$$

In (12),* D* denotes the number of apoptotic hepatocytes or dead hepatocytes, *r*_hn_ represents the rate of apoptotic hepatocytes killed by activated neutrophils, and *r*_ah_ represents the recovery rate of apoptotic hepatocytes. Receptor-ligand kinetics ([*D* ^*n*^]/[*D* ^*n*^ + *k*_*c*3_^*n*^])*N*_*f*_ *D* ^*∗*^ represents the amount of apoptotic hepatocytes that bind to activated neutrophils, with binding rate modeled as a Hill-type function [*D* ^*n*^]/[*D* ^*n*^ + *k*_*c*3_^*n*^]. Activated neutrophils have recently been found to kill apoptotic hepatocytes [67]. After neutrophils adhere to apoptotic hepatocytes, neutrophils release harmful chemical substances such as reactive oxygen species and proteases that accelerate death of apoptotic hepatocytes [67, 68]. When multiplying ([*D* ^*n*^]/[*D* ^*n*^ + *k*_*c*3_^*n*^])*N*_*f*_ *D* ^*∗*^ by *r*_hn_, the entire first term in (12) represents the number of apoptotic hepatocytes killed by activated neutrophils per hour, which is the total number of dead hepatocytes per hour. The maximum number of apoptotic or dead hepatocytes does not exceed the total number of hepatocytes in the liver (represented by *A*_*∞*_). In addition, *r*_ah_ represents the recovery rate of apoptotic hepatocytes, and the second term in (12) is defined as the amount of recovering apoptotic hepatocytes. The definition of parameters and corresponding experimental data for newly added system parameters in damaged tissue model are summarized in Table 3 (refer to Appendix).

### 2.5. Step 4: Monocyte Immune Response Model

Recent biological experiments from the literature [69, 70] have shown that monocyte, recruited by the presence of HMGB-1, significantly impacts liver inflammation and liver fibrosis. Upon liver injury, inflammatory Ly6cC (Gr1C) monocyte subset, as precursors of tissue macrophages in blood vessels near the infected site, is attracted and recruited to the injured liver via CCR2-dependent bone marrow egress. The chemokine receptor CCR2 and its ligand MCP-1/CCL2 promote monocyte subset infiltration upon liver injury and further promote the progression of liver fibrosis [26, 67]. Because evidence has shown that Tumor Necrosis Factor-*α* (TNF-*α*) induces a marked increase in CCL2/MCP-1 production in dose- and time-dependent manners [71], we assumed the influx of monocytes from blood vessels to liver is induced by effects of HMGB-1 and TNF-*α*. Therefore, we modeled the influx of monocytes similarly to kinetics of neutrophils influx. According to existing literature, HMGB-1 is released by necrotic cells and activated monocytes [22, 71, 72]. Therefore, we modeled the release of HMGB-1 using receptor-ligand kinetics and enzymatic kinetics, similar to the release of TNF-*α*, by incorporating effects of necrotic cells and activated monocytes:

(13) dPdt=kpgP1−PP∞−rpmkPnPn+kc1nMkfP∗−rpnPnPn+kc2nNf+NbP∗−rpmPnPn+kc4nMf+MbP∗,

(14) dNbdt=PnPn+kc2nNfP∗−umnNbMf∗−knubNb,

(15) dMRdt=kmrMR1−MRMs−r2MRH+T∗−μmrMR,

(16) dMfdt=r2MRH+T∗+kumbMb−PnPn+kc4nMfP∗−μmMf,

(17) dMbdt=PnPn+kc4nMfP∗−kumbMb,

(18) dHdt=rh1max⁡Mb+Dmh1+Mb+DMb+D−uhH.

In (13), we incorporate the effect of phagocytosis by monocytes into (4) because monocytes phagocytose pathogen by a CD14-dependent mechanism [73]. We recalled the Hill-type function equation ([*P* ^*n*^]/[*P* ^*n*^ + *k*_*c*4_^*n*^]) to represent receptor-ligand binding kinetics between pathogens and activated monocytes. Because binding activated neutrophils are engulfed by infiltrating monocytes [27], we used *u*_*mn*_ *N*_*b*_ *M*_*f*_^*∗*^ to calibrate the killing process of binding activated neutrophils by activated monocytes, thereby modifying (10) to (14). Equations (15), (16), and (17) describe activation and migration of resting monocytes from blood vessels to infected tissue. In (15), (16), and (17), *M*_*R*_, *M*_*f*_, and *M*_*b*_ represent resting monocytes, free activated monocytes, and binding activated monocytes, respectively. Principles used to build those three equations are similar to the principle used to build (8), (9), and (10) for the neutrophil immune response model. Equation (18) calibrates the release of HMGB-1 per hour by activated monocytes and apoptotic hepatocytes. The process of releasing HMGB-1 is similar to the process of releasing TNF-*α*. The definition of parameters and corresponding experimental data for newly added system parameters in the monocyte immune response model are summarized in Table 4 (refer to Appendix).

### 2.6. Step 5: SDMM of Innate Immunity

As one type of anti-inflammatory cytokines, IL-10 was found to prevent subsequent tissue damage by inhibiting activation of phagocytes, including neutrophils and monocytes [74]. This anti-inflammatory mediator, produced by macrophages, dendritic cells (DC), B cells, and various subsets of CD4+ and CD8+ T cells [75], follows the same mechanism as proinflammatory (TNF-*α* and HMGB-1) release. Because our main focus in this paper was to model innate immune responses, we ignored the release of IL-10 by B cells and T cells during adaptive immune responses; therefore, we modeled the release of IL-10 similarly to proinflammatory cytokine release:

(19) $$\begin{array}{c} \frac{dC_{A}}{dt}=\left(\;\frac{r_{ca\max}M_{b}}{C_{Ah}+M_{b}}\;\right)M_{b}-u_{ca}C_{A}. \end{array}$$

In (20), *C*_*A*_ represents the number of anti-inflammatory cytokine (IL-10) during AIR, and (*r*_*ca*max⁡_ *M*_*b*_/(*C*_*Ah*_ + *M*_*b*_)) represents the release rate of anti-inflammatory cytokine (IL-10) by activated monocytes, derived from enzymatic kinetics. The first term in (20) calibrates the increase in the number of anti-inflammatory cytokines every hour and the second term *u*_*ca*_ *C*_*A*_ calibrates the decrease in the number of anti-inflammatory cytokines every hour due to natural degradation. Corresponding parameters and their values are defined in Table 5 (refer to Appendix). After incorporating (*C*_*A*_, *x*) = *x*/(1 + *C*_*A*_/*C*_*∞*_), the inhibition function of IL-10, we derived a comprehensive mathematical model for innate immunity of AIR as follows. *C*_*∞*_ represents the dissociation rate of IL-10 with initial estimated value equivalent to 0.02. Consider

(20) dPdt=kpgP1−PP∞−rpmkPnPn+kc1nMkfP∗−rpnPnPn+kc2nNf+NbP∗−rpmPnPn+kc4nMf+MbP∗,

(21) dMkfdt=kmkMkf1−MkfK∞+kmkubMkb−PnPn+kc1nMkfP∗−umkMkf,

(22) dMkbdt=PnPn+kc1nMkfP∗−kmkubMkb,

(23) dTdt=rt1max⁡Mkbmt1+MkbMkb+rt2max⁡Nbmt2+NbNb−utT,

(24) dNRdt=krdNR1−NRNS−r1NRT+P∗1+CA/C∞−μnrNR,

(25) dNfdt=r1NRT+P∗1+CA/C∞+knubNb−PnPn+kc2nNfP∗−μnNf,

(26) dNbdt=PnPn+kc2nNfP∗−umnNbMf∗−knubNb,

(27) dr1dt=kr11+tanh⁡Nf∗−μr1r1,

(28) dDdt=rhnDnDn+kc3nNfD∗1−DA∞−rahD,

(29) dMRdt=kmrMR1−MRMs−r2MRH+T∗1+CA/C∞−μmrMR,

(30) dMfdt=r2MRH+T∗1+CA/C∞+kumbMb−PnPn+kc4nMfP∗−μmMf,

(31) dMbdt=PnPn+kc4nMfP∗−kumbMb,

(32) dHdt=rh1max⁡Mb+Dmh1+Mb+DMb+D−uhH,

(33) dCAdt=rcamax⁡MbCAh+MbMb−ucaCA.

In this 14-equation SDMM, variables *P*, *M*_*kf*_, *M*_*kb*_, *T*, *N*_*R*_, *N*_*f*_, *N*_*b*_, *r*_1_, *D*, *M*_*R*_, *M*_*f*_, *M*_*b*_, *H*, and *C*_*A*_ represent levels of pathogen, free Kupffer Cell, bound Kupffer Cell, TNF-*α*, resting neutrophil, free activated neutrophil, bound activated neutrophil, rate of resting neutrophil activated under infection, damaged tissue, resting monocyte, free activated monocytes, bound activated monocytes, HMGB-1, and IL-10, respectively. These variables are identified and selected as essential indicators in AIR. All system parameters (*k*_pg_ and so on), which reflect the strength of the host's immune system, are adjustable during model simulation. Detailed description of system parameters is presented in the Appendix.

### 2.7. Step 6: SDMM Incorporated with Adaptive Immunity

Innate immunity plays a significant role in regulating pathogen clearance through multiple types of cell interactions, providing the first line of defense during early stages of inflammation. Compared to innate immunity, adaptive immunity is typically recognized as a late stage of immune response to infection activated by antigen presenting cells (APCs) [76]. The nature of adaptive immune response is more complicated than innate immune responses and involves numerous interactions among cells and cytokines. To simplify adaptive immunity, we selected four representative cells, including CD4+ T cells, CD8+ T cells, B cells, and antibodies, to simulate a series of immune responses during pathogenic inflammation. The 18-equation SDMM incorporated with adaptive immunity is presented as follows:

(34) dPdt=kpgP1−PP∞−rpmkPnPn+kc1nMkfP∗−rpnPnPn+kc2nNf+NbP∗−rpmPnPn+kc4nMf+MbP∗−rpAbPnPn+kc5nAP∗−rpcd4PnPn+kc6nTCD4P∗,

(35) dMkfdt=kmkMkf1−MkfK∞+kmkubMkb−PnPn+kc1nMkfP∗−umkMkf,

(36) dMkbdt=PnPn+kc1nMkfP∗−kmkubMkb−rMkbcd8MkbnMkbn+kc7nTCD8Mkb∗,

(37) dTdt=rt1max⁡Mkbmt1+MkbMkb+rt2max⁡Nbmt2+NbNb−utT,

(38) dNRdt=krdNR1−NRNS−r1NRT+P∗1+CA/C∞−μnrNR,

(39) dNfdt=r1NRT+P∗1+CA/C∞+knubNb−PnPn+kc2nNfP∗−μnNf,

(40) dNbdt=PnPn+kc2nNfP∗−umnNbMf∗−knubNb−rNbcd8NbnNbn+kc7nTCD8Nb∗,

(41) dr1dt=kr11+tanh⁡Nf∗−μr1r1,

(42) dDdt=rhnDnDn+kc3nNfD∗1−DA∞−rahD,

(43) dMRdt=kmrMR1−MRMs−r2MRH+T+TCD4+TCD8∗1+CA/C∞−μmrMR,

(44) dMfdt=r2MRH+T+TCD4+TCD8∗1+CA/C∞+kumbMb−PnPn+kc4nMfP∗−μmMf,

(45) dMbdt=PnPn+kc4nMfP∗−kumbMb−rMbcd8MbnMbn+kc7nTCD8Mb∗,

(46) dHdt=rh1max⁡Mb+Dmh1+Mb+DMb+D−uhH,

(47) dCAdt=rcamax⁡MbCAh+MbMb−ucaCA,

(48) dTCD4dt=kcd4TCD41−TCD4Tcd4∞+rcd4MbMbnMbn+kc8nMb∗TCD4−kcd4MTcd4nTcd4n+kc10nTCD4∗Mb+Mf−ucd4TCD4,

(49) dTCD8dt=kcd8TCD81−TCD8Tcd8∞+rcd8MbMbnMbn+kc8nMb∗TCD8−kcd8MTcd8nTcd8n+kc10nTCD8∗Mb+Mf−ucd8TCD8,

(50) dBdt=kBB1−BB∞+rBtBnBn+kc9nB∗Tcd4−uBB,

(51) dAdt=rAbmax⁡BmAb+BB−uAbA.

Equation (48) describes the recruiting process of CD4+ T cells during adaptive immunity. The first term *k*_cd4_ *T*_CD4_(1 − *T*_CD4_/*T*_cd4*∞*_) in (48) is a standard logistic function to describe the natural migration process of CD4+ T cells to the site of infection, and *k*_cd4_ is a constant parameter to define the recruitment rate of CD4+ T cells from lymph node to the site of infection under undefined mechanisms in our SDMM. Activated monocytes that are phagocytizing pathogens were recognized as one type of APCs; APCs display major histocompatibility complex class II (MHCII) peptide on the surface available for binding to T cell antigen-specific receptor (TCR) [77]. APCs also activate the TCR on CD4+ T cells and enhance CD4+ T cell migration to the site of infection through a TCR-MCHII receptor-ligand response [76], represented by the second term, *r*_cd4*Mb*_(*M*_*b*_^*n*^/(*M*_*b*_^*n*^ + *k*_*c*8_^*n*^))*M*_*b*_^*∗*^ *T*_CD4_. Similar to the receptor-ligand response we modeled in innate immunity, we used a Hill-type (*M*_*b*_^*n*^/(*M*_*b*_^*n*^ + *k*_*c*8_^*n*^)) function to model the binding rate of activated monocytes to CD4+ T cells. Receptor-ligand kinetics *r*_cd4*Mb*_(*M*_*b*_^*n*^/(*M*_*b*_^*n*^ + *k*_*c*8_^*n*^))*M*_*b*_^*∗*^ *T*_CD4_ represent the amount of CD4+ T cells activated by activated monocytes. Our model assumes that T cells become activated under TCR-MCHII receptor-ligand response; however, we recognize that the activation process of T cells is much more complicated than we modeled because T cell activation requires at least two signals in order to become fully activated [77–80]. CD4+ T cells that undergo apoptosis are phagocytized by activated monocytes [81], represented by the third term in (48). We assume that free activated monocytes and binding activated monocytes phagocytize binding CD4+ T cells, represented by a receptor-ligand response *k*_cd4*M*_([*T*_cd4_^*n*^]/[*T*_cd4_^*n*^ + *k*_*c*10_^*n*^])*T*_CD4_^*∗*^(*M*_*b*_ + *M*_*f*_), with the binding rate equal to *k*_cd4*M*_([*T*_cd4_^*n*^]/[*T*_cd4_^*n*^ + *k*_*c*10_^*n*^]) and the phagocytosis rate equal to *k*_cd4*M*_. The fourth term, *u*_cd4_ *T*_CD4_, in (48) describes a natural apoptosis process of CD4+ T cell during migration and activation processes.

**Figure 3:**
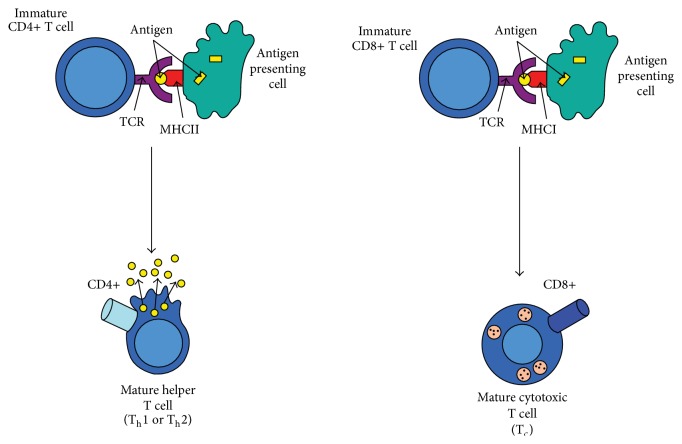
A simplified mechanism of T cell activation.

Similar to (48), (49) describes the recruitment process of CD8+ T cells during adaptive immunity. The activation process of CD8+ T cells through a major histocompatibility complex class I peptide- (MHCI-) TCR mechanism follows similar receptor-ligand kinetics of CD4+ T cells, represented by the second term, *r*_cd8*Mb*_(*M*_*b*_^*n*^/(*M*_*b*_^*n*^ + *k*_*c*8_^*n*^))*M*_*b*_^*∗*^ *T*_CD4_, in (49). The activation process of CD4+ T cells and CD8+ T cells is depicted in Figure 3.

CD4+ T and CD8+ T cells mediate the host response to sepsis in various ways. Experimental studies have shown that T_h_1 effector cells proliferated by CD4+ T cells can improve the phagocytosis rate of Kupffer Cells, activated neutrophils, and activated monocytes through a receptor-ligand response [82]. To simplify our SDMM, we used CD4+ T cell population to substitute for T_h_1 effector cell population, and we measured a decrease in the amount of pathogens via CD4+ T cell-dependent interactions using receptor-ligand kinetics, represented by the sixth term *r*_*p*cd4_([*P* ^*n*^]/[*P* ^*n*^ + *k*_*c*6_^*n*^])*T*_CD4_ *P* ^*∗*^ in (34). CD8+ T cells are cytotoxic cells because their primary function is to kill infected target cells [82, 83]. Therefore, we incorporated receptor-ligand kinetics into the third term in (36), the fourth term in (40), and the third term in (45) to measure the decrease in binding Kupffer Cells, binding activated neutrophils, and binding activated monocytes. In SDMM, we used the population of binding Kupffer Cells, binding activated neutrophils, and binding activated monocytes to represent the population of infected cells under the assumption that binding cells bind to pathogens. Therefore, the population of binding cells was also used to represent the population of APCs in our SDMM.

Macrophage activation is related to IFN-gamma released by T cells [84–86]. Because we did not calibrate IFN-gamma in our SDMM, we calculated the monocyte activation process using CD4+ T cell and CD8+ T cell populations instead of interferon-gamma (IFN-gamma) population for simplicity. Under this assumption, we revised the second term in (43) and the first term in (44) to *r*_2_ *M*_*R*_(*H* + *T* + *T*_CD4_ + *T*_CD8_)^*∗*^/(1 + *C*_*A*_/*C*_*∞*_). The newly revised term, *r*_2_ *M*_*R*_(*H* + *T* + *T*_CD4_ + *T*_CD8_)^*∗*^/(1 + *C*_*A*_/*C*_*∞*_), incorporates the CD4+ T cell and CD8+ T cell populations to reflect the role of CD4+ T cells and CD8+ T cells in the resting monocyte activation process.

T_h_1 or T_h_2 effector cells activate B cells to release antibodies [76]. Equation (50) describes the activation process of B cells by the CD4+ T cell population under the assumption that the CD4+ T cell population can represent T_h_1 and T_h_2 effector cell populations due to model simplification. The first term *k*_*B*_ *B*(1 − *B*/*B*_*∞*_) in (50) measures the migration process of B cells from lymph nodes to the site of infection, which is derived from a standard logistic function. Derivation of the second term, *r*_*Bt*_([*B* ^*n*^]/[*B* ^*n*^ + *k*_*c*9_^*n*^])*B* ^*∗*^ *T*_cd4_, in (50) is similar to derivation of the second terms in (48) and (49), following a receptor-ligand kinetics. Decrease in B cell population was induced by natural apoptosis, represented by the third term, *u*_*B*_ *B*, in (50). Plasma cells secrete antibodies [76], but we did not incorporate this specific mechanism into our SDMM. Instead, we modeled that antibodies were released by B cells. In (51), the release of antibodies from B cells is represented by the first term, (*r*_*Ab*max⁡_ *B*/(*m*_*Ab*_ + *B*))*B*, following receptor-ligand kinetics and enzymatic kinetics (Michaelis-Menten), similar to TNF-*α*, HMGB-1, and IL-10 release process described in innate immunity. The second term, *u*_*Ab*_ *A*, in (51) describes the natural catabolism of antibodies. When antibodies are released from plasmas cells, T_H_ cells define the isotype of the antibody [76]; we did not model specific isotype of antibodies in our model. Antibodies can opsonize pathogen and contribute to further pathogen clearance at the late stage of inflammation [76, 82], as represented by the fifth term, *r*_*pAb*_([*P* ^*n*^]/[*P* ^*n*^ + *k*_*c*5_^*n*^])*AP* ^*∗*^, in (34). The definition and corresponding experimental data for newly added system parameters in SDMM incorporated with adaptive immunity are summarized in Table 6 (refer to the Appendix).

## 3. Simulated Results

**Figure 4:**
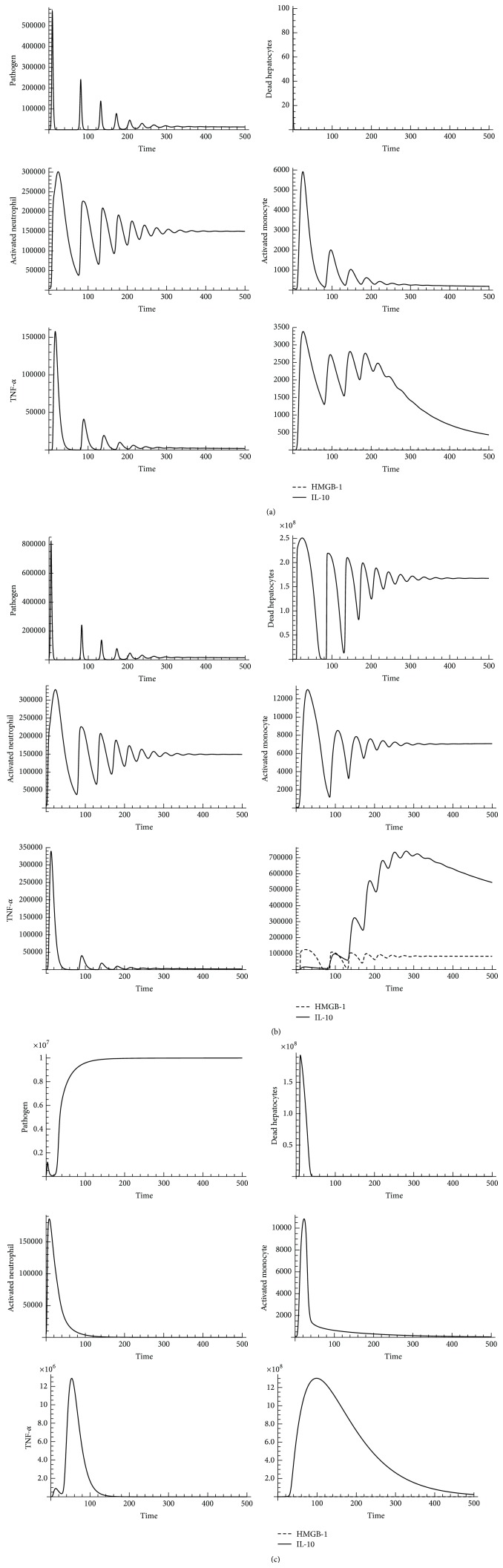
Dynamic patterns of AIR progression related to various initial levels of indicators and adjustable system parameters. *x*-axis represents time (in hours) and *y*-axis represents number of indicators (pathogen, dead hepatocyte, activated neutrophil, activated monocyte, TNF-*α*, HMGB-1, and IL-10) during AIR progression. (a) Combined dynamic patterns of indicators represent a* Healing Process* in AIR progression (pathogen initial counts = 100). (b) Combined dynamic patterns of indicators represent a* Persistent Infection* in AIR progression (pathogen initial counts = 10000). (c) Combined dynamic patterns of indicators represent* Organ Dysfunction* in AIR progression (pathogen initial counts = 100000).

Using SDMM, we identified three distinct dynamic patterns of indicators that represent three states of AIR progression:* Healing Process, Persistent Infection*, and* Organ Dysfunction*. Based on our computed results, we concluded that a* Healing Process* occurs when the level of pathogens, level of phagocytic cells (neutrophils and monocytes), and level of inflammatory cytokines (TNF-*α*, HMGB-1, and IL-10) oscillate below threshold during infection. We recognized that a* Persistent Infection *occurs if inflammatory responses are active (damaged tissue oscillates above threshold during infection). We also recognized that* Organ Dysfunction* occurs if an overwhelming load of bacteria is observed. Computed results are shown in Figure 4.

**Figure 5:**
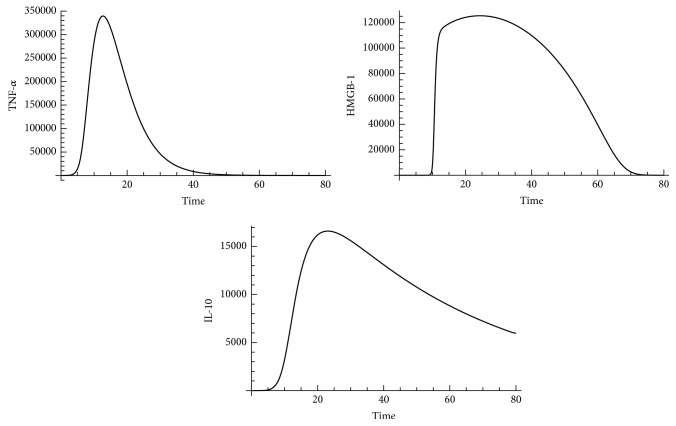
Dynamic patterns of TNF-*α*, HMGB-1, and IL-10 in mice livers during AIR generated from our SDMM. *x*-axis represents time (in hours) and *y*-axis represents number of indicators.

In order to initially validate our SDMM, model behaviors were compared to results from experimental designs under specific parameter settings. If results did not match, model reconfiguration was implemented by adjusting the relationship between components (indicators) or fine-tuning parameter values. We compared our simulated results to experimental results [87] and simulated results from a latest version of an AIR progression mathematical model [13]. We observed that our simulated results had better agreement with experimental results compared to simulated results from the previous mathematical model because our simulated results captured a dampened oscillated infection. We recognized that this improvement of simulation accuracy is a result of additional cellular and molecular pathways of AIR progression incorporated into our SDMM compared to previous mathematical models [12, 13]. For example, we simulated the effect of monocytes in our SDMM by incorporating interactions of monocytes with other cells and cytokines. In contrast, previous mathematical models simulated the combined effect of neutrophils and monocytes with the limitation of oversimplification of AIR progression. Our simulated results indicated that time required for peak levels of TNF-*α*, HMGB-1, and IL-10 is approximately 12 hrs, 18 hrs, and 24 hrs, respectively. These results are consistent with results from clinical trials [28], as shown in Figure 5.

We also explored the impact of pathogen initial load on phagocytic cells, inflammatory cytokines, and damaged tissue at low, medium, and high levels during AIR progression. We found that dynamic patterns of AIR progression were identified as “*Healing Process*” if the initial number of pathogens was set below 3.2 (result was transformed to a base-10 logarithm) in simulation; dynamic patterns of AIR progression were identified as “*Persistent Infection*” if the initial number of pathogens was set between 3.2 and 5.9 (result was transformed to a base-10 logarithm) in simulation; and dynamic patterns of AIR progression were identified as “*Organ Dysfunction*” if the initial number of pathogens was set above 5.9 (result was transformed to a base-10 logarithm) in simulation. During some simulation replications, our findings are inconsistent with pieces of evidence found from experimental studies [88–90] that indicated outcomes of AIR progression are more likely to lead to a healthy state with a low-dose of pathogens, which will be further illustrated in the Discussion.

**Figure 6:**
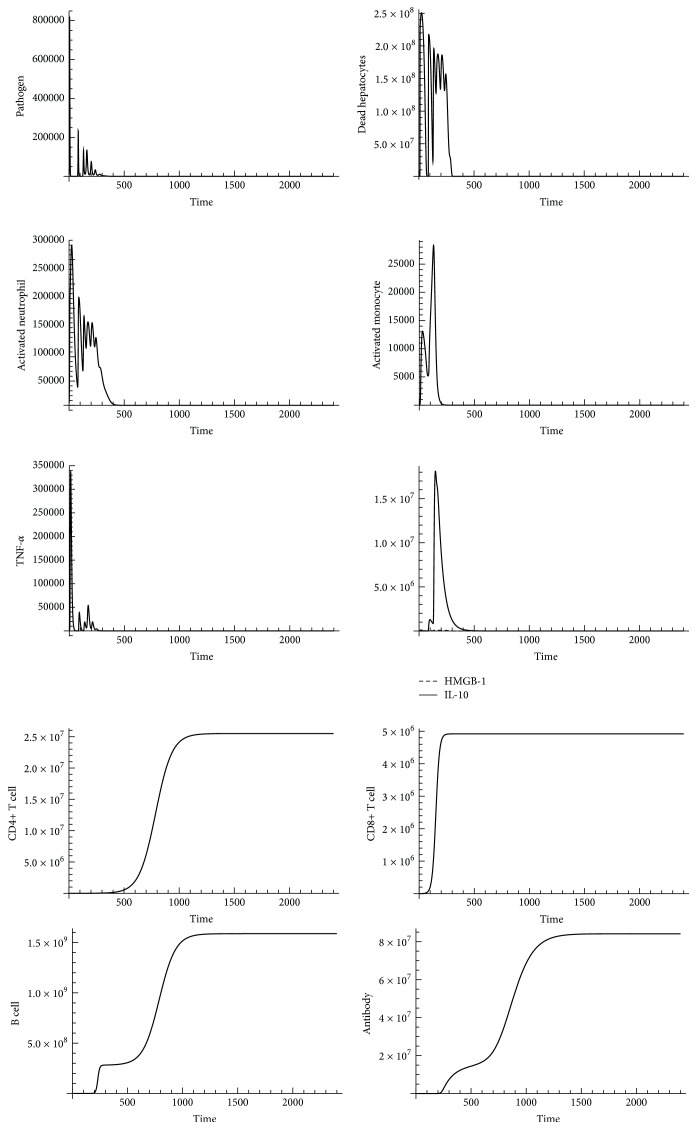
An adaptive immunity influence on outcomes of sepsis progression (pathogen initial counts = 10000). *x*-axis represents time (in hours) and *y*-axis represents number of indicators.

**Figure 7:**
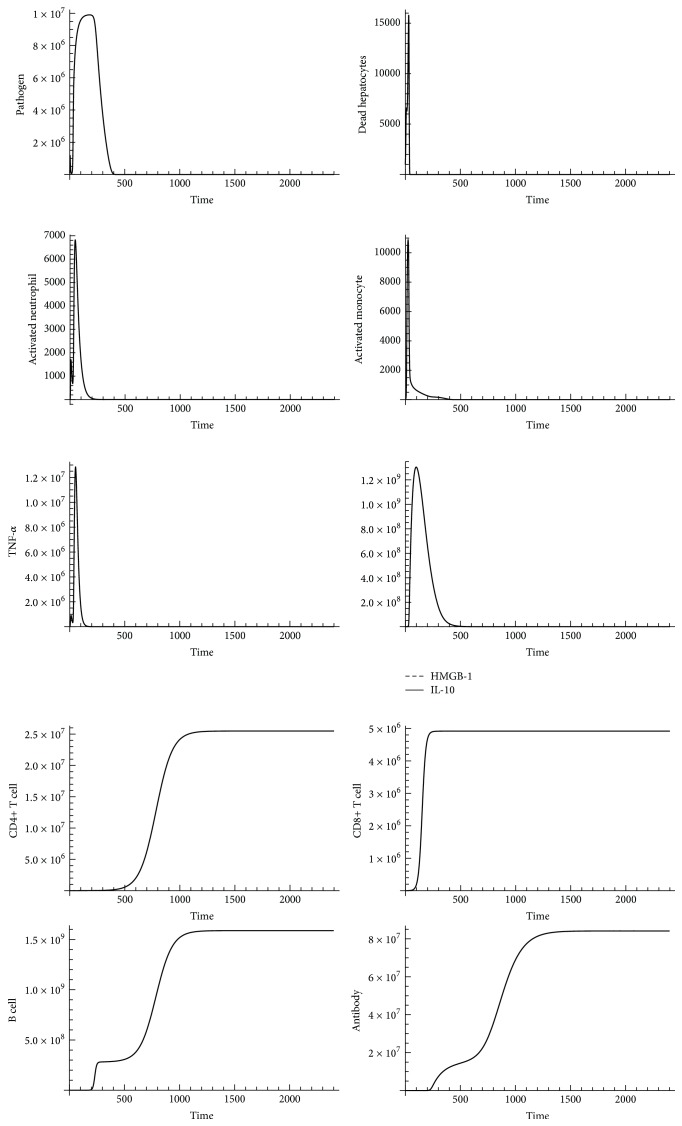
An adaptive immunity influence on outcomes of sepsis progression (pathogen initial counts = 100000). *x*-axis represents time (in hours) and *y*-axis represents number of indicators.

By incorporating adaptive immunity to SDMM, we generated dynamic patterns of pathogen count, dead hepatocyte count, activated neutrophil count, activated monocyte count, TNF-*α*, HMGB-1, IL-10, CD4+ T cell, CD+ 8 T cell, B cell, and antibodies using Mathematica (Wolfram Mathematica 9.0). Computed results are shown in Figures 6 and 7.

Based on our computed results, we observed pathogen count converged toward 0 at approximately 14 days (336 hrs) after infection during a* Persistent Infection* when the effect of adaptive immunity was incorporated into the full model. Compared to* Persistent Infection* observed in innate immunity (shown in Figure 4(b)), the activated neutrophil count and HMGB-1 count converged toward 0 at approximately 25 days (600 hrs) after infection. Convergence in TNF-*α* count occurred at approximately 14 days after infection, earlier than convergence in HMGB-1 count in innate immunity. The peak level of activated monocytes increased to 26000, which was 2 times higher than the peak level of activated monocytes observed in innate immunity. No additional dead hepatocytes were observed after 25 days (600 hrs) after infection because cells (activated neutrophils and activated monocytes) and cytokines (TNF-*α*, HMGB-1, and IL-10) associated with further tissue damage converged toward 0, indicating adaptive immunity positively impacted outcomes of sepsis progression.

By incorporating CD4+ T cells, CD8+ T cells, B cells, and antibodies into innate immunity, we observed that elevated pathogen count during* Organ Dysfunction* began to drop at approximately 20 days after infection (500 hrs), and the process of pathogen clearance induced by adaptive immunity persisted approximately 5 days after infection. Pathogen count returned to 0 at 25 days after infection (720 hrs). Cells (activated neutrophils and activated monocytes) and cytokines (TNF-*α*, HMGB-1, and IL-10) associated with innate immunity dropped significantly during simulation, but CD4+ T cells, CD8+ T cells, and B cells persistently elevated after 500 hrs after infection, indicating adaptive immunity's contribution to pathogen clearance during the late stage of sepsis progression. A mice model infected with a high dose of* Escherichia coli* [52] showed that the number of CD4+ T cells, CD8+ T cells, and B cells persisted throughout 7 days, thereby conforming to dynamic patterns of CD4+ T cells, CD8+ T cells, and B cells observed in our SDMM.

## 4. Stability Analysis

In order to study model behaviors under various parameter settings and initial conditions, bifurcation diagrams were used to conduct stability analysis for each subsystem during model construction. The objective of stability analysis was to identify key parameters or key processes in sepsis episodes. Numerical analysis that we used is similar to the previous study [16].

We started with stability analysis by calculating equilibrium points in Kupffer Cell local response model. The equilibrium points were derived by setting equations in Kupffer Cell local response model free of the time (time is denoted by *t* in equations), which imply that

(52) kpgP−1−P−P∞−rpmkP−nP−n+kc1nMkf− P−∗=0,

(53) kmkMkf−1−Mkf−K∞+kmkubMkb−−P−nP−n+kc1nMkf− P−∗−umkMkf−=0,

(54) P−nP−n+kc1nMkf− P−∗−kmkubMkb−=0.

To solve (52), (53), and (54), we firstly added (53) to (54), which eliminate the (53) and (54) to (55):

(55) $$\begin{array}{c} k_{mk}\overset{-}{M_{kf}}\left(\;1-\frac{\overset{-}{M_{kf}}}{K_{\infty }}\;\right)-u_{mk}\overset{-}{M_{kf}}=0. \end{array}$$

By solving (52) and (55) together, we could obtain the following feasible equilibrium points.

If *k*_*mkub*_ ≠ 0,

(56) $$\begin{array}{c} \left(\;\overset{-}{M_{kf}}=0,\overset{-}{P}=0,\overset{-}{M_{kb}}=0\;\right)\\ \text{or}\;\;\left(\;\overset{-}{M_{kf}}=0,\overset{-}{P}=P_{\infty },\overset{-}{M_{kb}}=0\;\right)\\ \text{or }\left(\;\overset{-}{M_{kf}}=\frac{k_{\infty }\left(k_{mk}-\mu_{mk}\right)}{k_{mk}},\overset{-}{P}=0,\overset{-}{M_{kb}}=0\;\right). \end{array}$$

The above equilibrium points are valid if the following conditions are satisfied:

(57) k∞≠0,P∞≠0,kc1≠0,n>0.

From the derived feasible equilibrium points, we obtained two disease-free equilibrium points given as

(58) $$\begin{array}{c} \left(\;\overset{-}{M_{kf}}=0,\overset{-}{P}=0,\overset{-}{M_{kb}}=0\;\right)\\ \text{or }\left(\;\overset{-}{M_{kf}}=\frac{k_{\infty }\left(k_{mk}-\mu_{mk}\right)}{k_{mk}},\overset{-}{P}=0,\overset{-}{M_{kb}}=0\;\right). \end{array}$$

We further calculated the associated Jacobian matrix to determine stability of the disease-free equilibrium points; the Jacobian matrix was given as follows:

(59) $$\begin{array}{c} \left[\begin{array}{ccc} k_{pg}-\frac{2k_{pg}\overset{-}{P}}{P_{\infty }}-\frac{r_{pmk}\overset{-}{M_{kf}}\sigma }{P_{\infty }}, & -r_{pmk}\frac{{\overset{-}{P}}^{n+1}}{\left({\overset{-}{P}}^{n}+k_{c1}^{n}\right)P_{\infty }}, & 0\\ -\frac{\overset{-}{M_{kf}}\sigma }{P_{\infty }}, & \left(k_{mk}-u_{mk}\right)-\frac{2k_{mk}\overset{-}{M_{kf}}}{K_{\infty }}-\frac{{\overset{-}{P}}^{n+1}}{\left({\overset{-}{P}}^{n}+k_{c1}^{n}\right)P_{\infty }}, & k_{mkub}\\ \frac{\overset{-}{M_{kf}}\sigma }{P_{\infty }} & \frac{{\overset{-}{P}}^{n+1}}{\left({\overset{-}{P}}^{n}+k_{c1}^{n}\right)P_{\infty }}, & -k_{mkub} \end{array}\right], \end{array}$$

where $\sigma =\left(n{\overset{-}{P}}^{2n}-n{\overset{-}{P}}^{n}\left({\overset{-}{P}}^{n}+k_{c1}^{n}\right)\right)/{\left({\overset{-}{P}}^{n}+k_{c1}^{n}\right)}^{2}$.

Replacing the first disease-free equilibrium point $\left(\overset{-}{M_{kf}}=0,\overset{-}{P}=0,\overset{-}{M_{kb}}=0\right)$ into the Jacobian matrix above (59), we can further derive the following Jacobian matrix:

(60) $$\begin{array}{c} J_{1}=\left[\begin{array}{ccc} k_{pg} & 0 & 0\\ 0 & k_{mk}-u_{mk} & k_{mkub}\\ 0 & 0 & -k_{mkub} \end{array}\right]. \end{array}$$

In order to find the associated eigenvalues with (60), we solved the following equation:

(61) det⁡J1−λI=kpg−λ000kmk−umk−λkmkub00−kmkub−λ=0.

Using Mathematica (Wolfram Mathematica 9.0), we obtained the eigenvalues of (61) as follows:

(62) λ11=−kmkub,λ21=kpg,λ31=kmk−umk.

Thus, we concluded that the first disease-free equilibrium point is stable if and only if the following conditions are satisfied:

(63) kmkub>0,kpg<0,kmk<umk.

Following a similar procedure above, we replaced the second disease-free equilibrium point $\left(\overset{-}{M_{kf}}=k_{\infty }\left(k_{mk}-\mu_{mk}\right)/k_{mk},\overset{-}{P}=0,\overset{-}{M_{kb}}=0\right)$ into the Jacobian matrix in (59).

The Jacobian matrix associated with the second disease-free equilibrium point was revised to

(64) $$\begin{array}{c} J_{2}=\left[\begin{array}{ccc} k_{pg} & 0 & 0\\ 0 & u_{mk}-k_{mk} & k_{mkub}\\ 0 & 0 & -k_{mkub} \end{array}\right]. \end{array}$$

Again, by solving (65),

(65) det⁡J2−λI=kpg−λ000umk−kmk−λkmkub00−kmkub−λ=0.

We obtained the eigenvalues associated with the second disease-free equilibrium point, and the eigenvalues were expressed as follows:

(66) λ12=−kmkub,λ22=kpg,λ32=umk−kmk.

Thus, the stability of the second disease-free equilibrium can be achieved if and only if the following conditions are satisfied:

(67) kmkub>0,kpg<0,kmk>umk.

Because *k*_pg_ (the growth rate of pathogen) was assumed to be always larger than 0, we concluded that the disease-free equilibrium points for Kupffer Cell local response model are always unstable.

**Figure 8:**
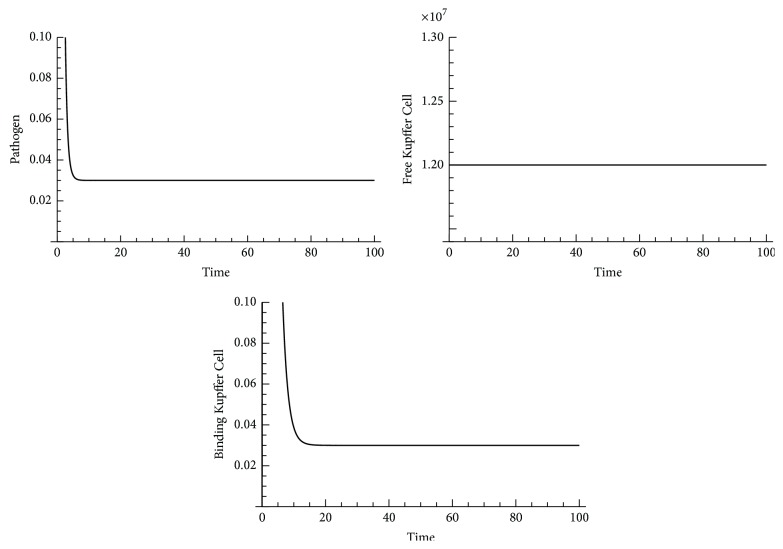
Change in the disease-free equilibrium point $\left(\overset{-}{M_{kf}}=12000000,\overset{-}{P}=0,\overset{-}{M_{kb}}=0\right)$ when* P* = 2 and *k*_pg_ = 1.2.

In order to verify our conclusion, we did a numerical study on the second disease-free equilibrium point $\left(\overset{-}{M_{kf}}=12000000,\overset{-}{P}=0,\overset{-}{M_{kb}}=0\right)$. We found the disease-free equilibrium point $\left(\overset{-}{M_{kf}}=12000000,\overset{-}{P}=0,\overset{-}{M_{kb}}=0\right)$ changed if pathogen load was changed from 0 to 2 at equilibria (a small perturbation was given); the simulated results of change in the disease-free equilibrium point $\left(\overset{-}{M_{kf}}=12000000,\overset{-}{P}=0,\overset{-}{M_{kb}}=0\right)$ are shown in Figure 8.

We also analyzed stability of the pathogen saturation equilibrium point $\left(\overset{-}{M_{kf}}=0,\overset{-}{P}=P_{\infty },\overset{-}{M_{kb}}=0\right)$. By numerical analysis, we concluded that the pathogen saturation equilibrium point $\left(\overset{-}{M_{kf}}=0,\overset{-}{P}=P_{\infty },\overset{-}{M_{kb}}=0\right)$ is stable if the following conditions are satisfied:

(68) kmk<0.5,umk>0.2.

**Figure 9:**
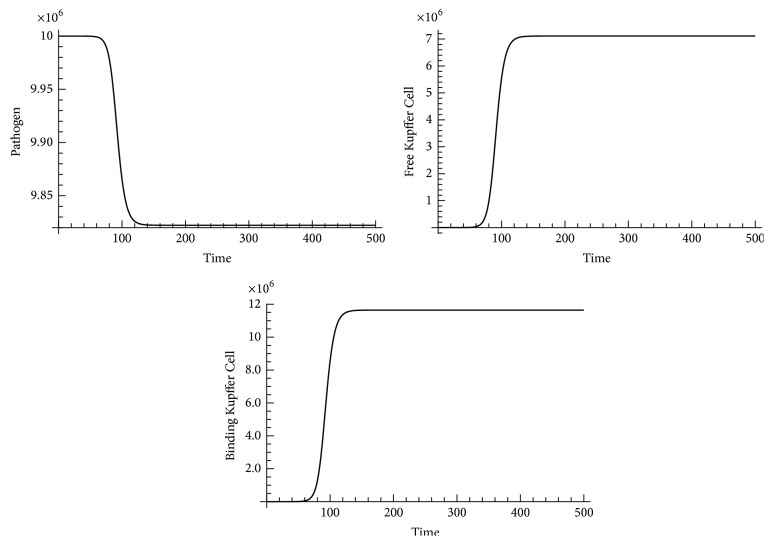
Change in the pathogen saturation equilibrium point $\left(\overset{-}{M_{kf}}=0,\overset{-}{P}=P_{\infty },\overset{-}{M_{kb}}=0\right)$ when *M*_*kf*_ = 2 and *k*_*mk*_ = 0.9.

When *k*_*mk*_ > 0.5, the pathogen saturation equilibrium point $\left(\overset{-}{M_{kf}}=0,\overset{-}{P}=P_{\infty },\overset{-}{M_{kb}}=0\right)$ became unstable. Simulated results of change in the pathogen saturation equilibrium point $\left(\overset{-}{M_{kf}}=0,\overset{-}{P}=P_{\infty },\overset{-}{M_{kb}}=0\right)$ are shown in Figure 9.

Stability analysis of equilibrium points in Kupffer Cell local response model indicated that Kupffer Cell local response model is not a stable system. The disease-free equilibrium point $\left(\overset{-}{M_{kf}}=12000000,\overset{-}{P}=0,\overset{-}{M_{kb}}=0\right)$ changed when the second infection occurred (*P* was changed from 0 to 2). However, recruiting more Kupffer Cells positively contributed to the pathogen clearance after a saturated infection (*P* = *P*_*∞*_), as shown in Figure 9.

Bifurcation diagrams are graphical tools to visualize dynamic system behavior changes with parameters. In this paper, we used Matcont to generate bifurcation diagrams. Matcont, a Matlab continuation package with a graphic user interface (GUI) for interactive numerical study of parameterized nonlinear ordinary differential equations (ODEs), computes curves of equilibria, limit points, Hopf point, limit cycles, fold, torus, and branch point bifurcation of limit cycles [91].

**Figure 10:**
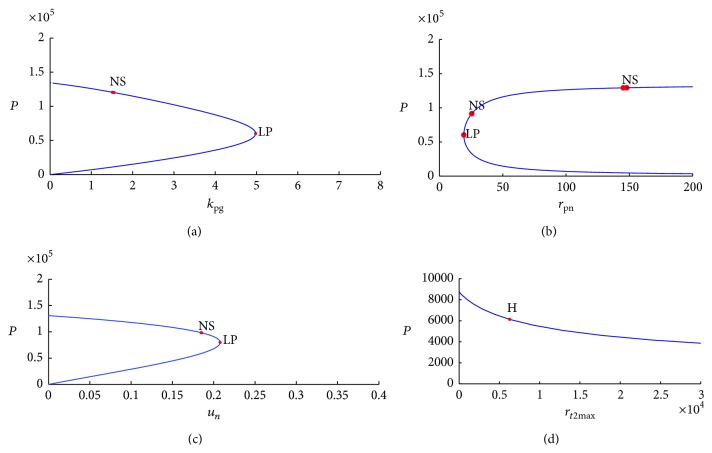
(a) Computed equilibrium curve of* pathogen *in relation to system parameter *k*_pg_ in neutrophil immune response model. (b) Computed equilibrium curve of* pathogen* in relation to system parameter *r*_*pn*_ in neutrophil immune response model. (c) Computed equilibrium curve of* pathogen* in relation to system parameter *u*_*n*_ in neutrophil immune response model. (d) Computed equilibrium curve of* pathogen* in relation to system parameter *r*_*t*2max⁡_ in neutrophil immune response model.

In bifurcation diagrams, *y*-axis represents equilibria of state variable and *x*-axis represents value of system parameter that generates equilibria. Therefore, bifurcation diagrams reflect change in equilibria of dynamic systems (change in number of equilibria or change in numerical value of equilibria) in relation to change in numerical value of system parameters. We analyzed stability of dynamic systems by identifying types of bifurcation points in bifurcation diagrams because bifurcation points are defined as points at which stability changes from stable to unstable. Two typical bifurcation points were evident in our bifurcation diagrams: limit point (marked as “LP” in Matcont) and Hopf point (marked as “H” in Matcont). Neutral saddle point was marked as “NS” in the bifurcation diagram, but it is not a bifurcation point for equilibrium because it is identified as a hyperbolic saddle.  Figure 10 shows that change in equilibria of state variable* pathogen* is related to change in system parameters in the neutrophil immune response model.

LPs in bifurcation diagrams of neutrophil immune response model appeared when two equilibria merged into one equilibrium; the number of equilibria of dynamic systems changed when LPs were detected. LPs are also turning points at which dynamic systems change from stability to instability. In Figure 10(a), stable equilibria of* pathogen* are observed when system parameter *k*_pg_ increases from 0 to 4.93. When *k*_pg_ equals 4.93, LP is identified and unstable equilibria of* pathogen* are generated as *k*_pg_ decreases from 4.93 to 0. Therefore, equilibria of* pathogen* of our neutrophil immune response model are bistable when *k*_pg_ ranges from 0 to 4.93. Similarly, equilibria of* pathogen* in Figure 10(b) are bistable when system parameter *r*_*pn*_ ranges from 25 to 200. In Figure 10(c), equilibria of* pathogen* are bistable when *u*_*n*_ ranges from 0 to 0.21.

**Figure 11:**
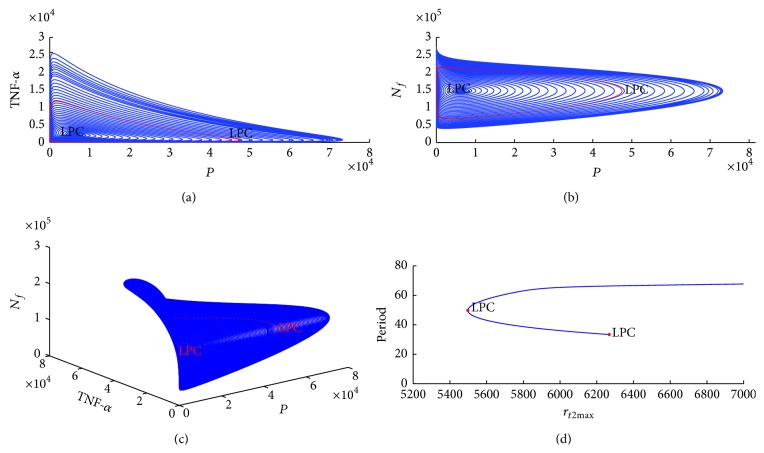
(a) Family of limit cycles bifurcating from the Hopf point in* TNF-a* and* pathogen* plane. (b) Family of limit cycles bifurcating from the Hopf point in *N*_*f*_ and* pathogen* plane. (c) Equilibria and limit cycles in (*N*_*f*_,* pathogen*, and* TNF-a*) space. (d) Period of the cycle as function of *r*_*t*2max⁡_.

A Hopf bifurcation, identified in Figure 10(d), is a periodic bifurcation in which a new limit cycle is born from a stationary solution. Hopf point, a turning point for periodic orbits, is detected when system parameter *r*_*t*2max⁡_ changes. The detected Hopf point in Figure 10(d) begins a limit cycle continuation in which two cycles collide and disappear. Because the first Lyapunov coefficient [92] is positive, an unstable limit cycle exists, bifurcating from this equilibrium. Figures 11(a) and 11(b) show the family of limit cycles bifurcating from detected Hopf point in Figure 10(d). The family of limit cycles is represented using limit cycle planes, such as* TNF-a*-*pathogen plane *and *N*_*f*_ *-pathogen* plane.  Figure 11(c) shows a limit cycle sphere represented by a* TNF-a*, *N*_*f*_, and* pathogen *plane.  Figure 11(d) indicates that two limit cycles occur when *r*_*t*2max⁡_ equals 5495.64 or 6265.00.

**Figure 12:**
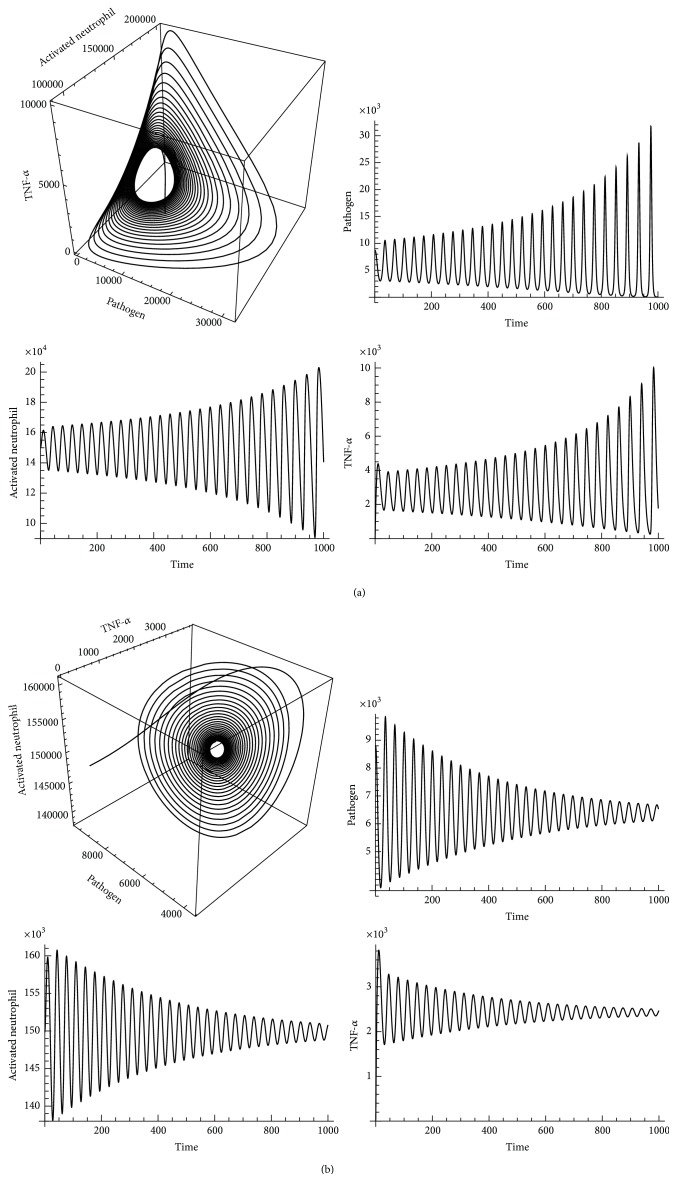
(a)* Pathogen*,* activated neutrophil,* and* TNF-α* diverge at unstable equilibria in neutrophil immune response model when *r*_*t*2max⁡_ is above 6265.00. (b)* Pathogen*,* activated neutrophil*, and* TNF-α* converge at stable equilibria in neutrophil immune response model when *r*_*t*2max⁡_ is between 5495.64 and 6265.00.

In Figure 11(c), the first family of limit cycle (small red cycle in the center of the sphere) spirals outward as system parameter *r*_*t*2max⁡_ decreases, and the second family of limit cycle appears when *r*_*t*2max⁡_ decreases to 5495.64 (a red cycle line appears). As *r*_*t*2max⁡_ increases from 5495.64, the second family of limit cycle spirals outward again. When *r*_*t*2max⁡_ increases to 6265.00, an unstable equilibrium is detected, as depicted in Figure 12(a). If value of *r*_*t*2max⁡_ is between 5495.64 and 6265.00, equilibria of the neutrophil immune response model are stable and converged, as shown in Figure 12(b). This finding infers either a high release rate of TNF-*α* (*r*_*t*2max⁡_ is above 6265.00) or a low release rate of TNF-*α* (*r*_*t*2max⁡_ is below 5495.64), thereby inducing generation of unstable equilibria in the neutrophil immune response model. From a biological response perspective, high release rate of TNF-*α* indicates overproduction of proinflammatory cytokines related to overwhelming proinflammation; low release rate of TNF-*α* leads to failure to recruit a sufficient amount of neutrophils related to infection clearance. Based on our stability analysis, we found that the release rate of TNF-*α* can positively or negatively influence outcomes of AIR progression, thereby conforming to experimental perturbation findings regarding effectiveness of anti-TNF-*α* therapies [93–95].

Continued stability analysis on the monocyte immune response model indicated that change in system parameters *k*_*rd*_, *u*_*nr*_, and *u*_*n*_ induces bistability of the monocyte immune response model. We observed that the monocyte immune response model was bistable if at least one of the following three conditions was met: *k*_*rd*_ was between 0 and 0.32, *u*_*nr*_ was between 0 and 0.28, or *u*_*n*_ was between 0 and 0.21. Specifically, we observed that *r*_*t*2max⁡_ (maximum release rate of TNF-*α* by activated neutrophil) and* m*_*t2*_ (number of activated neutrophils at which the reaction rate is half of the maximum production rate) are essential for oscillated monocyte immune response model. Similar to the neutrophil immune response model, limit cycles bifurcate from Hopf point. Therefore, we conclude that the oscillated infection is dependent on the amount of released TNF-a and recruited neutrophils in AIR progression. However, released monocytes and associated cytokines such as HMGB-1 do not contribute to oscillation in AIR progression.

Building upon the monocyte immune response model, we incorporated the effect of anti-inflammatory cytokine (IL-10) into the full model. We observed that Hopf point was detected when *r*_*t*2max⁡_ increased to 128000 because anti-inflammatory cytokine inhibited activation of phagocytic cells (neutrophils and monocytes). This trend indicates that infection oscillation (harmful outcomes) requires additional proinflammation activated by neutrophils in the full model, compared to monocyte immune response model without including the effect of anti-inflammatory cytokine. Therefore, our simulated results demonstrated that AIR progression is more likely to end with* Healing Process* if the effect of anti-inflammatory cytokine is incorporated.

Strengthened (increased *r*_*t*2max⁡_ and* m*_*t2*_) proinflammatory immune responses could also induce stable or unstable equilibria, leading to a dampened oscillated infection or diverged infection, similar to our observations in Figure 12. However, we observed that if high effect of anti-inflammatory cytokine was incorporated (dissociation rate equal to a base-10 logarithm 8) at the beginning of infection, AIR progression resulted in an unstable overwhelming pathogen load at equilibria (refer to Figure 12(a)). However, a stable dampened oscillated pathogen load at equilibria (refer to Figure 12(b)) was observed if medium effect of anti-inflammatory cytokine (dissociation rate equal to a base-10 logarithm 5) was incorporated. These observations confirmed that effects of anti-inflammatory cytokine can be positive or negative to AIR progression depending on levels of anti-inflammatory cytokine.

**Figure 13:**
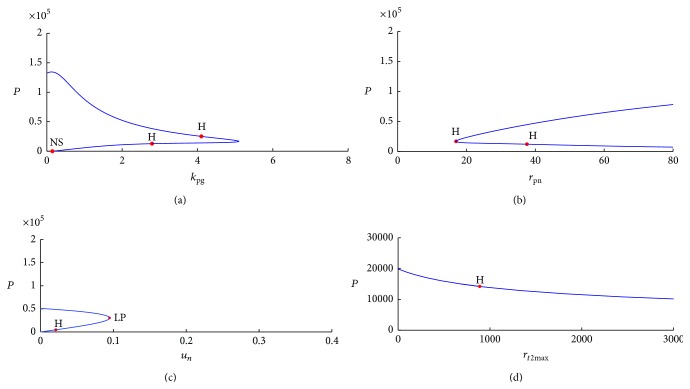
(a) Computed equilibrium curve of* pathogen *in relation to system parameter *k*_pg_ in the full model incorporated with adaptive immunity. (b) Computed equilibrium curve of* pathogen* in relation to system parameters *r*_*pn*_ in the full model incorporated with adaptive immunity. (c) Computed equilibrium curve of* pathogen* in relation to system parameters *u*_*n*_ in the full model incorporated with adaptive immunity. (d) Computed equilibrium curve of* pathogen* in relation to system parameters *r*_*t*2max⁡_ in the full model incorporated with adaptive immunity.

We conducted bifurcation analysis for the model incorporated with adaptive immunity, similar to bifurcation analysis we conducted in the neutrophil subsystem, monocytes subsystem, and full model. We selected four bifurcation diagrams as shown in Figure 13.

As shown in Figure 13(a), two Hopf bifurcations were detected at *k*_pg_ = 2.8 and *k*_pg_ = 4.1. Similarly, Hopf bifurcations were also detected in Figures 13(b) and 13(c) when *r*_*pn*_ = 17, *r*_*pn*_= 38, or *u*_*n*_ = 0.047. Compared to innate immunity, incorporation of adaptive immunity induced a further stabilized limit cycles, bifurcation from the equilibrium. Our stability analysis shown in Figure 13(d) illustrates that the Hopf bifurcation moves to lower *r*_*t*2max⁡_ value compared to Hopf bifurcation detected in innate immunity. The change in bifurcations indicated the contribution of adaptive immunity to sepsis progression.

**Figure 14:**
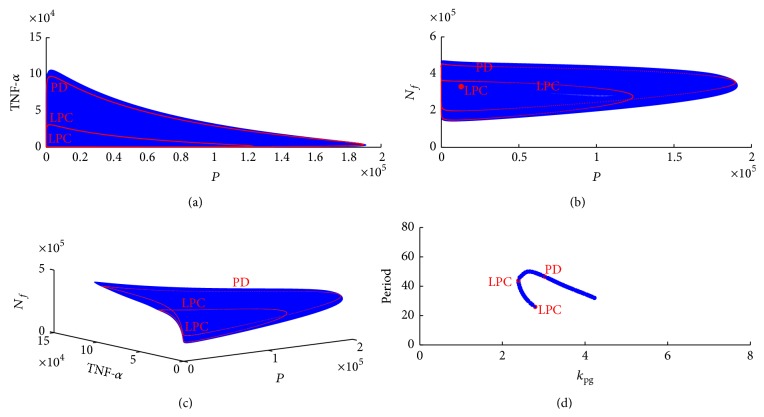
(a) Family of limit cycles bifurcation from the Hopf point (*k*_pg_ = 2.8) in* TNF-α* and* pathogen* plane. (b) Family of limit cycles bifurcating from the Hopf point (*k*_pg_ = 2.8) in *N*_*f*_ and* pathogen* plane. (c) Equilibria and limit cycles in (*N*_*f*_,* pathogen*, and* TNF-α*) space. (d) Period of the cycle as function of *k*_pg_.

In Figures 14(a), 14(b), and 14(c), the first family of limit cycle (small red cycle in the center of the sphere, marked as LPC) spiral outward as system parameter *k*_pg_ decreases, and the second family of limit cycle appears when *k*_pg_ decreases to 2.4 (a red cycle line appears). As *k*_pg_ increases from 2.4, the second family of limit cycle spirals outward again. A period doubling is detected when *k*_pg_ increases to 3, marked as PD in Figure 14. Because the first Lyapunov coefficient is negative, limit cycle bifurcations from the equilibrium are stable compared to unstable limit cycles detected in the neutrophil subsystem.

## 5. Discussion

Experimental results in literature have suggested that anti-inflammatory mediator inhibits activation of phagocytes and reduces the ability of activatedphagocytes to attack pathogen [96], consequently related to mortality and severity of infection in sepsis [97, 98]. However, other experimental studies have shown that anti-inflammatory cytokine downregulates production of secreted cytokines by inhibiting various behaviors of activated immune cells, thereby reducing the risk of tissue damage [28, 99, 100]. Our computed results from SDMM suggested that the effect of anti-inflammatory cytokines could be a “double-edged sword” for AIR because anti-inflammatory cytokine would either decrease mortality associated with tissue damage or increase mortality associated with high load of bacteria. With a low effect of anti-inflammatory cytokine (dissociation rate equal to a base-10 logarithm 2), our computed results showed that anti-inflammatory cytokine fails to inhibit the release of activated immune cells (activated neutrophils and activated monocytes) and subsequent cytokine production. Levels of damaged tissue significantly accumulated during the first 500 hours (approximately 20 days) of simulation. With the high effect of anti-inflammatory cytokine (dissociation rate equal to a base-10 logarithm 8), our simulated results and stability analysis demonstrated that sepsis progression leads to increased chance of death caused by overwhelming pathogen load at the end of simulation.

To further investigate effects of anti-inflammatory cytokines, we simulated a medium effect of anti-inflammatory cytokine (dissociation rate equal to a base-10 logarithm 5) and compared simulated results to high effect of anti-inflammatory cytokine and low effect of anti-inflammatory cytokine. Our computed results showed that pathogen load decreases during the first 100 hours of infection in combination with the total amount of dead hepatocytes. Furthermore, we observed that production of activated neutrophils and activated monocytes declined to baseline near 0 at the end of simulation, indicating a positive trend of sepsis progression to a healthy pattern. Therefore, we conclude that the level of anti-inflammatory cytokines significantly impacts direction of sepsis progression. We also conclude that levels of anti-inflammatory cytokine and time of intervention of anti-inflammatory cytokines determine outcomes of AIR under specific system configuration. Based on simulated results from our SDMM, we inferred that the survival rate of the host (chance of ending with a* Healing Process*) could be improved if a medium level of IL-10 injection was set between 3 hrs and 6 hrs after infection.

We assert that care must be taken when applying simulated results to clinics before the implementer fully understands the underlying setting of the simulation. Because it was difficult to simultaneously incorporate every intermediate biological process of inflammatory response, reasonable assumptions must be made when building a mathematical model. In our SDMM, we did not model* Salmonella* replicating within neutrophils. However, experimental study [58] asserted that neutrophils and macrophages were the primary sites for* Salmonella* proliferation in a mouse. Therefore,* Salmonella* replication could be considered in the future model if additional literature supported this fact. Various T cell types were reported to be able to express IL-10 under various conditions [101]. Therefore, IL-10 production estimation is difficult because IL-10 levels produced by T cells were various due to stimuli type or the strength of stimuli. In our model, we did not differentiate helper T cells from specific types that are identified in biological process. Plasma cells secrete antibodies [76], but we did not incorporate this specific mechanism in our SDMM. Instead, we modeled that B cells released antibodies. When antibodies are released from plasmas cells, T_H_ cells define the isotype of the antibody [76]; however, we also did not model specific isotype of antibody in our model. Furthermore, we ignored the fact that antibody opsonization induces stimulation of cytokine release when they are phagocytized by inflammatory cells. We ignored the fact that antibody opsonization induces stimulation of the release of various cytokines from neutrophils and macrophages [76]. Also, we ignored effects of other proinflammatory cytokines such as IL-1, IL-12, and IL-8 in our SDMM. Biological immune responses responding to infection are recognized as a series of complex processes including intracellular transductions (transfer of DNA) and intercellular pathways between cells. These biological processes will be developed with evolved understanding and continued investigation of cellular and molecular mechanisms [34], which could be further research interests in the field. In our SDMM, we used numerical count, or the number of indicators in the simulation, as the estimate of cell or cytokine number in AIR. In practice, physicians must translate data to measurable units of indicators similar to how we translated clinical data to simulation data. Furthermore, our conclusion regarding IL-10 was drawn based on specific simulation settings including setting system parameters and initial loads of indicators. Initial system setting must be fully understood before considering application of IL-10 level for preclinic experiments.

Based on our simulated results regarding anti-inflammatory cytokine, we propose a hypothesis testing: If medium levels of anti-inflammatory antibody were injected into the host with sepsis between 3 hrs and 6 hrs would survival rates of the host improve under hyperinflammation? The purpose of this hypothesis testing is to detect effective zones of the anti-inflammatory antibody related to* Healing Process *of AIR in order to help develop therapeutic agents in preclinical trials.

According to our simulation study, we found that initial levels of pathogen significantly impact dynamic patterns of AIR progression. However, inconsistency in observations between our simulated results and existing experimental studies forces us to propose another hypothesis testing: What is the range of initial loads in pathogen with a maximum likelihood of leading to a* Healing Process*? After discussing with experts in the field, our initial assumption is that if the initial load of pathogen is low, AIR progression has a chance to end with a* Persistent Infection* because immune responses fail to be fully activated at the beginning of infection. However, if the initial load of pathogen is high, the immune system fails to control and regulate infection that could also lead to* Organ Dysfunction*. The purpose of this hypothesis testing is to detect dangerous zones of initial loads in pathogens in order to develop effective therapeutic targets in preclinical trials.

Mathematical modeling, at various levels, could regulate individual components of inflammation and provide insights into biological interactions in order to understand complex inflammatory processes during sepsis progression. However, the traditional mathematical model has unique disadvantages. First, the model fails to capture stochastic process for heterogeneous populations. Second, the model fails to describe local interactions between heterogeneous populations, such as the movement of tissue macrophage towards the local pathogen in the infected area. In order to improve simulation accuracy and overcome disadvantages of the mathematical model, a hybrid modeling framework may be used to model and simulate sepsis progression in future research [102].

## 6. Conclusion and Future Research

We proposed an 18-equation system dynamic mathematical model and showed that the model has significant potential to predict possible pathogenesis of sepsis based on the host's physiological conditions. Also, we showed that the model provides essential biological insight into innate immunity and adaptive immunity of sepsis episodes by exploring various combinations of phagocyte and cytokine levels. We focused primarily on the combined effects of pathogen load, phagocytic cells, tissue damage, anti-inflammatory cytokine, CD4+ T cell, CD8+ T cell, B cell, and antibodies by adding cellular pathways during sepsis progression. We observed that outcomes of sepsis progression could be improved with IL-10 at a medium level in an early stage of infection (between the first 3 hrs and the first 6 hrs after infection). Furthermore, our model quantitatively measured levels of phagocytes (neutrophils and monocytes) and captured a* dampened oscillated infection* in AIR progression, compared to existing mathematical models that provide more accurate qualitative estimates.

Adaptive immunity contributes to further pathogen clearance after innate immunity because it includes B cells, T cells, and antibodies released from B cells [103]. We conducted an initial study of adaptive immunity during sepsis progression by incorporating CD4+ T cells, CD8+ T cells, B cells, and antibodies to the SDMM. We observed that CD4+ T cell count, CD8+ T cell count, B cell count, and antibody count were persistently elevated, which contributed to the pathogen clearance during a late stage of sepsis progression. Because we did not specify T cell type during SDMM, IL-10 production by T cells was not considered in the current SDMM. IL-10 production by T cells potentially leads to overproduction of anti-inflammatory cytokines by compensatory anti-inflammatory response and eventually increases risk of secondary infection and inaccurate prognosis [75, 103]. For further research, we expect to explore prominent effects of anti-inflammatory mediators secreted by T cells as they relate to outcomes of sepsis progression.

The system dynamic mathematical model proposed in this paper is a robust, accurate representation of comprehensive immune responses within a sepsis episode. This underlying model is general and flexible to be used to predict possible outcomes and prognosis for various hosts' initial conditions with various model parameters using experimental data from the literature. In addition, hypothesis testing proposed based on our simulated results could be a reference to help reduce unnecessary clinical trials and focus on essential processes of sepsis.

## Figures and Tables

**Table 1:** Definition of parameters and experimental values in *Kupffer Cell local response model*.

Parameters	Description	Value	References
*k*_pg_	*Salmonella* growth rate	1.2–3.6/h	[20]
*P*_*∞*_	*Salmonella* carrying capacity	10^8^ cells/g liver	[32]
*r*_*pmk*_	Rate at which pathogens are killed by Kupffer Cells	0.03/per Kupffer Cell/h	[31]
*n*	The extent of ligands binding to receptors (not specified)	2	Estimated
*k*_*c*1_	Concentration of Kupffer Cells which phagocytose half of *Salmonella *	0.03	[31]
*k*_*mk*_	Proliferation rate of Kupffer Cells under inflammation	0.015–2/h	Estimated
*K*_*∞*_	Kupffer Cell carrying capacity in liver	$\frac{(16\text{–}20)\times 10^{6}\;\;\text{cells}}{\text{g liver}}$	[40]
*k*_*mkub*_	Unbinding rate of binding Kupffer Cells	0.1–0.77/h	[33]
*u*_*mk*_	Killing rate of free Kupffer Cells induced by binding to pathogens	0.23–0.9/h	[33]

**Table 2:** Definition of parameters and experimental values in *neutrophil immune response model*.

Parameters	Description	Value	References
*r*_*pn*_	Rate at which pathogens are killed by neutrophils	20–100/per neutrophil/h	[41]
*r*_*t*1max⁡_	The maximum number of TNF-*α* being released by Kupffer Cells per enzyme molecule per hour	10/h	Estimated
*r*_*t*2max⁡_	The maximum number of TNF-*α* being released by activated neutrophils per enzyme molecule per hour	1000/h	Estimated
*m*_*t*1_	Number of Kupffer Cells at which the reaction rate is half of maximum production rate	10000 cells	Estimated
*m*_*t*2_	Number of activated neutrophils at which the reaction rate is half of maximum production rate	10000 cells	Estimated
*k*_*c*2_	Concentration of neutrophils which phagocytose half of *Salmonella *	1.5 × 10^−4^	[42]
*u*_*t*_	Degradation rate of TNF-*α*	0.025–0.5/h (measured in kidney)	[43]
*k*_*rd*_	Influx rate of neutrophils into blood vessel	0.1–0.72/h	[44]
*N*_*s*_	Resting neutrophil carrying capacity in blood vessel	3.5 × 10^5^ cells	[22]
*μ*_*nr*_	Apoptotic rate of resting neutrophils per hour	0.069–0.12/h	[45]
*μ*_*n*_	Apoptotic rate of activated neutrophils per hour	0.05/h	[45]
*k*_*nub*_	Unbinding rate of activated neutrophils per hour	0.01–0.5/h	Estimated
*k*_*r*1_	Auxiliary parameter associated with the activation rate of resting neutrophils	3/h	Estimated
*u*_*r*1_	Degradation rate of parameter *r*_1_ to maintain a slow-saturation curve	0.003/h	Estimated

**Table 3:** Definition of parameters and experimental values in *damaged tissue model*.

Parameters	Description	Value	References
*A*_*∞*_	Number of hepatocytes in liver	3.2 × 10^8^ cells	Mouse phenome database
*r*_hn_	Rate at which activated neutrophils kill apoptotic hepatocytes	9000/per neutrophil/h	Estimated
*k*_*c*3_	Concentration of activated neutrophils which phagocytose half of apoptotic hepatocytes	0.04	Estimated
*r*_ah_	Recovery rate of apoptotic hepatocytes	0.5–2/h	[46]

**Table 4:** Definition of parameters and experimental values in *monocyte immune response model*.

Parameters	Description	Value	References
*k*_*mr*_	Influx rate of monocytes into blood vessel	0.5/h	[44]
*r*_*pm*_	Rate at which pathogens are killed by inflammatory monocytes	7/per monocyte/h	[47]
*r*_2_	Influx rate of monocytes in liver	80/h	[48]
*M*_*s*_	Resting monocyte carrying capacity in blood vessel	50000 cells	[49]
*μ*_*mr*_	Apoptotic rate of resting monocytes per hour	0.2/h	Estimated
*μ*_*m*_	Apoptotic rate of activated monocytes (monocytes-derived-macrophage) per hour	0.08/h	[50]
*r*_*h*1max⁡_	The maximum number of HMGB-1 being released by monocytes per enzyme molecule per hour	0.001/h	Estimated
*m*_*h*1_	Number of monocytes at which the reaction rate is half of maximum production rate	10000 cells	Estimated
*k*_*c*4_	Concentration of monocytes which phagocytose half of *Salmonella *	0.002	[47]
*k*_*umb*_	Unbinding rate of binding activated monocytes	0.4/h	[51]
*u*_*h*_	Degradation rate of HMGB-1	0.5–3/h	Estimated
*u*_*mn*_	Rate at which activated neutrophils are killed by inflammatory monocytes	200/monocyte/h	Estimated

**Table 5:** Definition of parameters and experimental values in *full model*.

Parameters	Description	Value	References
*r*_*ca*max⁡_	The maximum number of IL-10 being released by monocytes per enzyme molecule per hour	10000/h	Estimated
*C*_*Ah*_	Number of monocytes at which the reaction rate is half of the maximum production rate	10000 cells	Estimated
*u*_*ca*_	Degradation rate of IL-10	0.02/h	Estimated
*C*_*∞*_	Dissociation rate of IL-10	0.02	Estimated

**Table 6:** Definition of parameters and experimental values in *full model with adaptive immunity*.

Parameters	Description	Value	References
*k*_cd4_	The influx rate of CD4+ T cells to blood vessel	0.014	[52]
*T*_cd4*∞*_	CD4+ T cell carrying capacity in the blood vessel	27.4 × 10^6^	[52]
*u*_cd4_	Degradation rate of CD4+ T cells	0.00083–0.001	[52]
*k*_cd8_	The influx rate of CD8+ T cells to blood vessel	0.0625	[52]
*T*_cd8*∞*_	CD8+ T cell carrying capacity in the blood vessel	5 × 10^6^	[52]
*u*_cd8_	Degradation rate of CD8+ T cells	0.00079–0.001	[52]
*k*_*B*_	The influx rate of B cells to blood vessel	0.0122	[52]
*B*_*∞*_	B cell carrying capacity in the blood vessel	28.6 × 10^6^	[52]
*u*_*B*_	Degradation rate of B cells	0.00012–0.00016	[53, 54]
*r*_*Ab*max⁡_	The maximum production amount of antibody by B cells	0.00053	[49, 55, 56]
*m*_*Ab*_	Number of B cells at which the reaction rate is half of maximum production rate	10000	Estimated
*u*_*Ab*_	Degradation rate of antibody	0.0035–0.01	[57]
*r*_*pAb*_	Rate at which pathogens are killed by antibody	1	Estimated based on [49, 56, 57]
*k*_*c*5_	Concentration of antibody which kills half of *Salmonella *	0.035	Estimated
*r*_*p*cd4_	Rate at which pathogens are killed by CD4+ T cells	8	[47, 58, 59]
*k*_*c*6_	Concentration of CD4+ T cells which kill half of *Salmonella *	0.0015	Estimated
*r*_*Mkb*cd8_	Rate at which binding Kupffer Cells are killed by CD8+ T cells	0.25	[59]
*k*_*c*7_	Concentration of CD8+ T cells which kill half of binding antigen presenting cells	0.0015	Estimated
*r*_*Nb*cd8_	Rate at which binding activated neutrophils are killed by CD8+ T cells	0.25	[59]
*r*_*Mb*cd8_	Rate at which binding activated monocytes are killed by CD8+ T cells	0.25	[59]
*r*_cd4*Mb*_	Rate at which CD4+ T cells bind to activated monocytes	4	[59]
*r*_cd8*Mb*_	Rate at which CD8+ T cells bind to activated monocytes	4	[59]
*k*_*c*8_	Activated monocyte concentration produces half occupation on T cells	0.0075	Estimated
*r*_*Bt*_	Rate at which B cells bind to T cells	1–10	Estimated
*k*_*c*9_	B cell concentration produces half occupation on T cells	0.045	Estimated
*k*_cd4*M*_	Rate at which binding CD4+ T cells are killed by activated monocytes	0.73–2	[60]
*k*_cd8*M*_	Rate at which binding CD8+ T cells are killed by activated monocytes	0.73–2	[60]
*k*_*c*10_	Concentration of activated monocytes which kill half of binding T cells	0.018	Estimated

## Appendix

See Tables 1–6.
